# Influence of consumer socio-psychological food environment on food choice and its implications for nutrition: evidence from Tanzania

**DOI:** 10.3389/fnut.2025.1589492

**Published:** 2025-08-25

**Authors:** Eward Mushi, Roselyne Alphonce, Betty Waized, Mikidadi Muhanga, Niloofar Khalili, Constance Rybak

**Affiliations:** ^1^Department of Agricultural Economics and Agribusiness, Sokoine University of Agriculture, Morogoro, Tanzania; ^2^Department of Development and Strategic Studies, Sokoine University of Agriculture, Morogoro, Tanzania; ^3^Leibniz-Centre for Agricultural Landscape Research (ZALF), Muncheberg, Germany; ^4^Thaer-Institute-Div. Urban Plant Ecophysiology, Humboldt-Universität zu Berlin, Berlin, Germany

**Keywords:** attitude, background factors, food choices, food environment, knowledge, perceived behavior control, Tanzania

## Abstract

**Background:**

Changes in consumer food choices have been associated with transformation in the food environment. Despite the direct impact of consumers' food choices on their diet and health outcomes, there is a lack of comprehensive evidence regarding how various factors within the food environment impact these choices.

**Methods:**

This study uses the Theory of Planned Behavior to examine how socio-psychological factors in the food environment influence consumers' healthy food choices. A survey of 714 randomly selected adult consumers was conducted in selected rural and urban areas in Tanzania. Data was analyzed using structural equation modeling.

**Results:**

The results indicate that consumers' knowledge, attitudes, and perceived behavioral control significantly explain 93% of the variance in the intention to choose healthy foods. The intention to choose healthy foods was found to be positive and significantly associated with the consumption of fruits and vegetables. Moreover, background factors, namely age, gender, location, and income, significantly influence the intention and actual behavior of healthy eating. Notably, the intentions of female and urban consumers to choose healthy foods do not converge with their consumption patterns of fruits and vegetables.

**Conclusion:**

These findings indicate that socio-psychological factors within the personal food environment are key determinants of food choice behavior. Thus, integrating consumers' socio-psychology into the food environment, as well as nutrition-sensitive and nutrition-specific interventions, can enhance consumers' knowledge of food choice and foster positive attitudes and perceptions regarding healthy eating. Food and health literacy programs could serve as effective strategies for achieving healthy eating behavior.

## 1 Introduction

Consumers' food choices directly impact their dietary quality, nutrition, and health outcomes. Food choices involve what, when, where, with whom, and how much to eat (1). It refers to the “people's thoughts, feelings, and actions related to food and eating” (2). Currently, many countries are experiencing a shift in food consumption patterns, with a growing preference for ultra-processed foods that are energy-dense, low in micronutrients, high in added sugar and salt, and often prepared outside the home, while the intake of healthier foods, such as fruits and vegetables, remains low (3–5). These changes in food choices, coupled with inadequate physical activity, are called nutrition transition, which is associated with overweight, obesity, and the resulting non-communicable diseases.

While the impact of the nutrition transition is a global challenge, low- and middle-income countries (LMICs), such as Tanzania, are particularly vulnerable due to the rapid transformation of their food systems (6). In many developed countries, there is a rising prevalence of overweight and obesity. In contrast, in most LMICs, there is a co-existence of overweight/obesity, wasting, stunting, and micronutrient deficiency (6), prompting increased attention from researchers and policymakers regarding key drivers of consumers' food choices. However, analyzing food choice is complex because it is associated with a range of interrelated factors including food itself, the person, and the economic, physical, biological, psychological, and socio-cultural factors (7–9, 60, 62, 69). According to the food systems conceptual framework proposed by the High-Level Panel of Experts on Food Security and Nutrition (HLPE) (63), these factors interact with the food environment. This has garnered increased attention among researchers and practitioners within the fields of agriculture, nutrition, and health, as the food environment serves as the interface through which the food system influences food choices (4, 10–12). However, there remains a lack of robust evidence to establish that interaction, underscoring the need for further research. The food environment refers to the interface where consumers interact with the broader food system to acquire food. According to Turner et al. (13), this interface includes the consumer, along with various food sources, such as formal and informal markets, food production, and food transfers. The food environment encompasses both external and personal domains. The external domains consist of factors beyond the consumer's control that influence food acquisition, including food availability, prices, vendor and product characteristics, and promotional information. Personal dimensions involve individual-level factors such as accessibility, affordability, convenience, and food desirability (3, 13). The personal food environment encompasses aspects such as the consumer's culture, psychology (including beliefs, attitudes, and perceptions), knowledge and skills, and social and demographic information related to food and eating (3, 10, 14).

Extensive research on the food environment has predominantly focused on the external dimensions (3, 11, 15, 58, 59, 67), with less attention given to aspects of the personal food environment, such as consumer psychology and demographic factors. Previous studies that have addressed psychological factors suggest that variables such as expectations regarding the satiating capacity of food (16), emotional states like sadness (17), perceived behavioral control and self-efficacy (18–20), as well as knowledge of and attitudes toward healthy eating (61), influence food choices.

Despite the valuable contributions of previous studies in understanding the influence of the personal food environment on food choices, there remains a need for further evidence to address the following issues. First, the literature on the food environment has primarily focused on developed countries (21–25), and its findings cannot be directly applied to the context of LMICs like Tanzania. This limitation stems from differences in the food environments, as well as variations in how consumers understand healthy foods across their countries, cultures, environments, personalities, and other social aspects (1, 20, 26). Additionally, social media, food promotions, and advertisements play a pivotal role in shaping consumers' attitudes and perceptions toward food, thereby influencing their food choices (27–29).

Third, the majority of studies on the personal food environment have focused on consumer psychology (30–32), particularly in relation to the intention to consume certain types of food, thereby creating the intention-behavior gap. According to Ajzen (33), one of the reasons for the intention–behavior gap is either forgetting to carry out the intended action or deciding to change one's mind about performing it. Thus, it is necessary to examine whether the behavioral intention is translated into performance. Fourth, most studies have reported inconsistent influences of socioeconomic and demographic factors on food choices (34, 35), pointing to a need for more context-specific evidence.

This study expands the understanding of how the personal food environment influences consumers' food choices in Tanzania by exploring the role of consumer sociopsychology through the lens of the Theory of Planned Behavior. Specifically, the study investigates (i) the influence of consumers‘ perceptions, attitudes, perceived behavioral control, and knowledge of healthy eating on the intention, and actual consumption of healthy foods, and (ii) the influence of the consumer's age, education, income, gender, location, and employment status on consumption of healthy foods. The findings are anticipated to equip policymakers with valuable insights to guide the development of policies and interventions to reverse the current nutrition transition.

## 2 Theoretical framework and hypotheses

Since the consumer's bounded rationality constrains the basic economic theory of consumer behavior, integrating behavioral economics and/or socio-psychological theories into food choice analysis can provide a deeper understanding of consumer behavior (11, 36, 37, 68). To that end, the Theory of Planned Behavior (TPB) was used in this study to capture the consumer's psychological behavior toward food choice. The TPB is used to predict an individual's intention to perform a particular behavior (38). According to the TPB, a person's intention to perform a certain behavior is determined by the person's attitude, knowledge, perceived behavior control, and subjective norms (38, 39, 64). These unobserved (latent) variables can influence behavioral intention directly or through mediation (64). Ajzen (33) adds that the TPB has background factors that may indirectly influence intention and behavior, or any element of the TPB. These background factors include socioeconomic and demographic factors, such as age and gender. This study uses the variables of the TPB along with background factors to establish its hypotheses.

Behavioral attitude is a person's general evaluation of the good or bad of performing a certain behavior (66). With this, we hypothesize that the consumer's attitude (*X*_1_) toward food significantly and positively predicts healthy food choice intention (γ_*i*_) (Equation 1). Specifically, consumers who perceive eating nutritious foods as beneficial will form the intention to choose those foods.


(1) $$\begin{array}{l} \gamma_{i}=\beta_{0}+\beta_{1}X_{1i}+\varepsilon .\gamma \end{array}$$

where β_1_ is the effect of *X*_1_ on γ_*i*_, and ε.γ is the error term for γ_*i*_.

Perceived behavioral control refers to people's perception of the ease or difficulty of performing a given behavior, and it is determined by external (resources and opportunities) and internal (ability, skills, and information) factors (38, 64, 74). We hypothesize that perceived behavioral control (*X*_2_) significantly and positively predicts consumers' intention to choose healthy foods (γ_*i*_) (Equation 2). Specifically, consumers with positive perceptions of their ability to access and prepare healthy foods are more likely to form intentions to choose healthy diets.


(2) $$\begin{array}{l} \gamma_{i}=\beta_{0}+\beta_{2}X_{2i}+\varepsilon .\gamma \end{array}$$

where β_2_ is the effect of *X*_2_ on γ_*i*_, and ε.γ is the error term for γ_*i*_.

Subjective norms refer to the person's external influence from people considered important in one's life on performing a given behavior (64). We hypothesize that consumers' subjective norms (*X*_3_) positively and significantly predict the intention to choose healthy foods (γ_*i*_) (Equation 3). Consumers who believe that others would support them in consuming healthy foods are more likely to develop intentions to eat healthily.


(3) $$\begin{array}{l} \gamma_{i}=\beta_{0}+\beta_{3}X_{3i}+\varepsilon .\gamma \end{array}$$

where β_3_ is the effect of *X*_3_ on γ_*i*_, and ε.γ is the error term for γ_*i*_.

Knowledge about healthy foods encompasses a consumer's ability to understand and interpret dietary information, skills in food preparation, awareness of the components of a balanced diet, and an understanding of their nutritional value and health benefits. Thus, we hypothesize that consumers' knowledge about healthy foods (*X*_4_) significantly predicts their intention to choose a healthy diet (γ_*i*_) (Equation 4). Specifically, greater knowledge about the benefits and attributes of healthy foods is associated with an increase in their intention to make healthier dietary choices.


(4) $$\begin{array}{l} \gamma_{i}=\beta_{0}+\beta_{4}X_{4i}+\varepsilon .\gamma \end{array}$$

where β_4_ is the effect of *X*_4_ on γ_*i*_, and ε.γ is the error term for γ_*i*_.

The structural models and hypotheses for the indirect prediction of the intention to choose healthy foods among the exogenous and endogenous latent variables of the TPB can be presented as follows:

Knowledge about healthy food significantly fosters a positive attitude toward eating a healthy diet (Equation 5).


(5) $$\begin{array}{l} X_{1i}=\beta_{0}+\beta_{4}X_{4i}+\varepsilon .X_{1} \end{array}$$

where β_4_ is the effect of *X*_4_ on *X*_1_, and ε.*X*_1_ is the error term for *X*_1_.

Knowledge about healthy food significantly influences perceived behavioral control (Equation 6). That is, the consumers' good knowledge about the importance of healthy eating is associated with the perception of easiness of forming healthy eating behavior.


(6) $$\begin{array}{l} X_{2i}=\beta_{0}+\beta_{4}X_{4i}+\varepsilon .X_{2} \end{array}$$

where β_4_ is the effect of *X*_4_ on *X*_2_, and ε.*X*_2_ is the error term for *X*_2_.

According to Ajzen (38), the general rule is that the stronger the intention to engage in a behavior, the more likely it is to be its performance. Therefore, we hypothesize that the consumer's positive intention to choose healthy foods is associated with the actual consumption of healthy foods (Equation 7).


(7) $$\begin{array}{l} \psi =\alpha_{0}+\alpha_{1}\gamma_{i}+\varepsilon .\psi \end{array}$$

where ψ is the behavior performance, which is the actual consumption of healthy, α_1_ is the coefficient showing whether the intention is translated into actual consumption of healthy foods, and ε.ψ is the error term for ψ.

Finally, on the background factors of the TPB, we hypothesize that the consumer's age, education, income, gender, location, and employment status significantly influence the consumption of healthy foods (Equation 8).


(8) $$\begin{array}{r} \psi =\Omega_{0}+\Omega_{1}Age+\Omega_{2}Education+\Omega_{3}Income+\Omega_{4}Location\\ +\Omega_{5}Gender\;\Omega_{6}HH\;Size+\;\Omega_{7}Employment\;Status+\varepsilon .\psi \end{array}$$

where Ω_1_ − Ω_7_ are coefficients estimating the influence of socioeconomic and demographic factors on the consumption of healthy foods.

## 3 Research methodology

### 3.1 Study design

**Figure 1:**
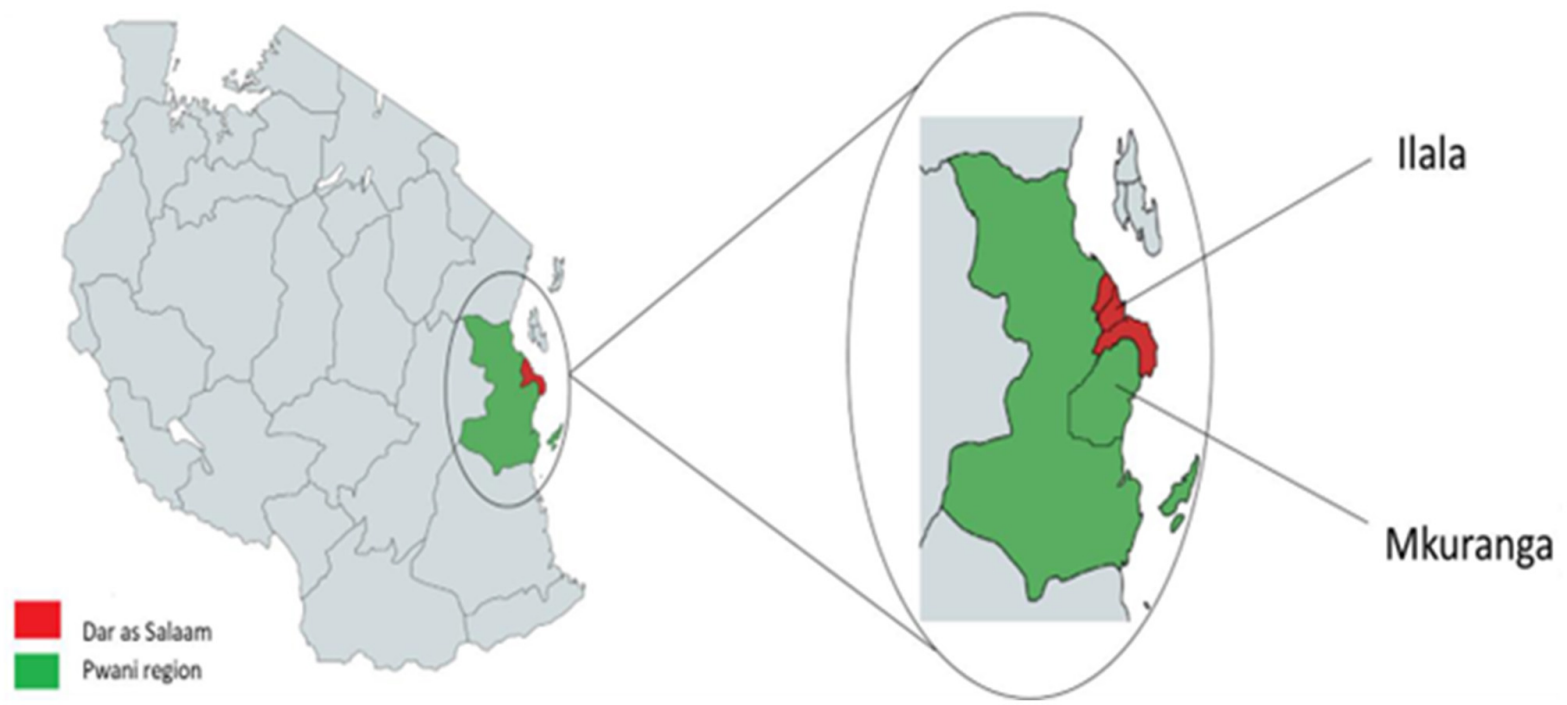
Map of the study area.

This study employed a cross-sectional research design (40), and data were collected in February 2023 using a structured questionnaire. The survey was part of the FoCo-Active project, which examines food consumption patterns among pupils and their parents in selected rural and urban areas of Tanzania. Specifically, the study was implemented in the Ilala and Mkuranga districts of Dar es Salaam and Pwani regions, respectively, along Tanzania's coastal zone (Figure 1). Dar es Salaam, the country's most cosmopolitan city with a population of 5,383,728, was purposefully chosen to represent urban settings, while Pwani, a peri-urban region with a population of 2,024,947, was chosen to represent rural consumers (41). Ilala, at the heart of Dar es Salaam city, is characterized by diverse economic activities, including manufacturing, retail and wholesale trade, hospitality services, food vending, banking, transportation, and urban agriculture. These activities shape its food environment, dominated by formal and informal markets. In contrast, Mkuranga's economy is largely driven by agriculture, including crop farming, fishing, and livestock keeping, which contributes to its natural and cultivated food environments (23), typifying the rural settings in Tanzania. Although both districts share similar agroecological conditions, with an average annual rainfall of 1,000 mm and average temperatures ranging from 27°C to 29°C (42), which suggests comparable food production and supply, the distinct food environments in each district may influence the consumers' food choices.

The data collected were based on the variables of the TPB. Since the variables are latent, specific indicators for each were developed through various research-based statements. Consumers had to indicate their perceptions of each statement through a seven-point Likert scale, and thus, for each construct, a mean score could be calculated. Approximately 40 indicators were used to measure the latent variables. These indicators were developed based on previous studies (31, 43, 44). Except for indicators of the constructs “cues of action” and “intention to consume healthy food,” which were measured on a 7-point Likert scale (1 = Definitely not, 7 = Definitely), indicators for the rest of the constructs were measured on a scale of 1 = Strongly disagree, 7 = Strongly agree.

The consumer's healthy food choice was captured through the actual consumption frequency of fruits and vegetables based on a 7-day recall period for food group consumption. Respondents were asked to indicate how many days in the past seven days they consumed food from a specific food group. Drawing on the definition provided by Deshpande et al. (70), we define healthy eating as a dietary pattern characterized by low-density lipoprotein (LDL), low levels of saturated fat and sugar, high fiber intake, and a high consumption of fresh fruits and vegetables. Despite the cultural, environmental, personal, and social differences in interpreting healthy food, fruits and vegetables are agreed to be the main components of a healthy diet (20, 26). Thus, in this study, the consumption frequency of fruits and vegetables is a proxy for healthy food choices.

### 3.2 Sampling procedures

A five-stage sampling procedure with stratification (45) was employed to randomly select 520 participants from Ilala District and 194 from Mkuranga District. The required sample size was determined using the formula in Equation 1, and the sample size allocation for the two study sites followed the principle of proportionality in size (45). In the first stage, the study sites, Ilala and Mkuranga districts, were purposively selected as representative urban and rural strata, respectively.


(9) $$\begin{array}{l} n=\frac{N}{1+N{\left(e\right)}^{2}} \end{array}$$

where *n* is the desired minimum sample size, *N* is the target population, and e is the margin of error (46). The target population was 3,970 households, and the margin of error used was 0.05. A 50% buffer was added to the computed sample size to accommodate the potential attrition. In the second stage, wards were purposively selected from each stratum. Three wards, namely Gongolamboto, Upanga, and Kinyerezi, were chosen from the Ilala district, while Mkamba and Kisegese were selected from the Mkuranga district. The wards' selection process in the Ilala district was designed to ensure adequate representation of middle- and high-socioeconomic status (SES) groups, while in Mkuranga, the focus was on selecting wards that typify rural Tanzanian communities. In the third stage, eight primary schools within the target population in each ward were selected proportional to their sizes. Respectively, six and two schools represented the Ilala and Mkuranga districts. Primary schools were used as a convenient method to create a sampling frame for parents, the target population for this study. In the fourth stage, pupils from the selected schools were chosen randomly, ensuring size proportionality in the allocation of the sample across schools. Finally, the adult representatives of the households of the selected pupils were contacted and included in the study sample of the 714 participants.

### 3.3 Data analysis

Descriptive statistics were employed to analyze the respondents' socio-economic characteristics and their responses to the indicators of the TPB variables. Moreover, to have a better understanding of the respondents' socioeconomic status (SES), the principal component analysis (PCA) was used to classify respondents into low, middle, and high SES. PCA is a statistical method used to reduce the complexity of a dataset by decreasing its dimensions (47). In this study, the variables used in the calculation of the SES index include monthly income, education level, employment status, type of toilet, water source, and possession of such assets as a house and refrigerator.

The structural equation modeling (SEM) technique was used to test the hypotheses of the first objective of this study, as specified by equations one to seven. The SEM, a method for estimating relationships of systems of latent variables, is considered a powerful multivariate approach because it is capable of testing both direct and indirect hypothesized systems of causal relationships (48, 72, 73). SEM has two components, namely the measurement model and the structural model. The former assesses the relationship between indicators and latent variables, while the latter assesses the causal relationship among the latent variables (65). Alongside the relationship of the TPB variables, the structural model specification was informed by a correlation analysis, which was carried out to identify and understand potential associations between the constructs, which helps to make correct path specification.

The measurement model was validated by performing validity and reliability analysis. The validity of the latent variables was assessed by average variance extracted (AVE) and composite reliability (CR). These indices evaluate the degree of confidence that a latent variable is well measured by its indicators (convergent validity) and the degree to which indicators of different latent variables are not related [discriminant/construct validity; (48, 65)]. Reliability was assessed using Cronbach's alpha coefficient and composite reliability (CR) to evaluate the internal consistency of the indicators in measuring the latent variables (49, 75).

Appropriate fitness indices used to test the model goodness of fit were Information Criterion (AIC), Swartz's Bayesian Information Criterion (BIC), Coefficient of Determination (*R*^2^), Root Mean Square Error of Approximation (RMSEA), Comparative Fit Index (CFI), Tucker-Lewis Index (TLI), and Standardized Root Mean Square Residual (SRMR). Most studies using SEM have applied at least some of these fit indices [see, for example, (48, 50, 74–76)]. These indices are important in determining whether the proposed models or theories fit the sample data (51).

The second objective of this study utilized a multivariate regression model to examine the impact of respondents' age, education, employment status, income, location of residence, gender, and household size on the choice of healthy foods. A multivariate regression model was found appropriate for the analysis because the food choices involved more than one food group (40). The model specification for this objective is provided in Equation 8.

## 4 Results and discussion

### 4.1 Characteristics of respondents

The results in Table 1 show that the majority (86%) of the interviewed respondents were female individuals, and in terms of geographical location, 73% of respondents were from urban areas. The education status of respondents indicates that over 80% of them have at least completed primary education, which indicates that most of them can at least read, write, and count. However, although most rural and urban respondents reported having completed primary school (>50%), a notable proportion (30%) of rural respondents reported having no formal education, which underscores the importance of considering the rural–urban divide in consumer analyses. In terms of income, the results of this study indicate that the overall annual median income is TZS 6,936,207, with rural households reporting a relatively larger proportion. The household size in the study area is about six people, while the average age of the respondents is 37.28 years, which indicates the presence of an active labor force.

**Table 1:** Characteristics of respondents.

**Categorical variables (*N* = 714)**	**Categories**	**Percentage**		
		**Pooled**	**Rural**	**Urban**
Sex	Male	14	25.32	10.02
	Female	86	74.68	89.98
Marital status	Married	76.99	81.16	75.64
	Not married	23.01	18.84	24.36
Education	No education	12.26	29.87	5.62
	Incomplete primary education	5.51	10.39	3.67
	Complete primary education	57.19	51.30	59.41
	Secondary education and above	25.04	8.44	31.30
Socio-Economic Status (SES)	Low SES	40	84.06	25.76
	Middle SES	20	15.22	21.55
	High SES	40	0.72	52.69
**Non-categorical variables**	**Unit of measurement**	**Mean (std. error)**		
Age of respondent (*N* = 714)	Years	37.28 (0.46)	41.7 (1.19)	35.93 (0.44)
Annual median income (*N* = 565)	TZS	6,936,207 (320,969.5)	5,075,266 (445,150.4)	7,507,048 (391,482.2)
Household size (*N* = 565)	Number of household members	5.59 (2.22)	6.4 (0.2)	5.33 (0.1)

Moreover, the PCA results show an equal proportion (40%) of respondents falling within the low SES and those in the high SES, while the remaining 20% are in the middle SES. However, the proportion structure of the SES classification differs significantly between rural and urban areas. Over 80% of rural respondents fall under the low SES, while over 50% of their urban counterparts were found to be in the high SES. These findings emphasize that despite some studies, such as FAO et al. (6) showing the rural–urban divide is declining regarding food consumption patterns, this SES divergence could pose a significant challenge to consumers' food choices. This divergence between rural and urban respondents suggests that it is important for the food environment interventions to consider the socio-economic disparities between the urban and rural communities.

### 4.2 Descriptive statistics for latent variables and their indicators

The mean scores of constructs that indicate the respondents' level of agreement/disagreement with the statements (indicators) relating to healthy food choices are presented in Table 2. The findings reveal that on a scale of 1–7 points, respondents are in high agreement with the statements of the perceived behavior control (mean score = 5.6), followed by knowledge of healthy eating (mean score = 5.4), and attitude toward healthy eating (5.2). This suggests that respondents generally agree (mean score > 5) with the statements that measure constructs of the TPB. The highest score on the perceived behavior control implies that respondents believe it is relatively easy for them to choose to consume healthy foods. The perceived ease in choosing healthy foods could be attributed to the consumer's ability and skills in food choices, and the available resources and opportunities to access healthy foods (74). The highest level of agreement is on the statement, “I should make sure I eat healthy so that I have the energy to take care of my family” (mean score = 5.64), suggesting that consumers understand the importance of making healthy food choices.

**Table 2:** Results of the measurement model.

**Constructs and their indicators**	**Mean score**	**Std. dev**.	**Factor loadings**	**Cronbach's alpha**	**Average variance extracted (AVE)**	**Composite reliability (CR)**
**Knowledge of healthy food**	**5.3730**	**1.4822**		**0.9098**	**0.7189**	**0.9108**
I understand that healthy food contains a lot of vitamins, minerals, and proteins	5.2011	1.5393	0.7902			
I understand that our health status is a result of what we choose to eat	5.3704	1.4969	0.8525			
Fruits and vegetables are important components of a healthy diet	5.5679	1.3986	0.8965			
Choosing to eat healthy foods makes me able to effectively participate in economic activities	5.3527	1.4941	0.8489			
**Attitude toward healthy eating**	**5.3143**	**1.1488**		**0.8936**	**0.6630**	**0.8861**
The food we choose to eat affects our nutritional status	5.3439	1.4438	0.7574			
Eating diverse food makes our bodies healthy	5.4286	1.4185	0.8834			
Eating fruits and vegetables makes our bodies healthy	5.5503	1.3943	0.9006			
Eating healthy foods can improve our economic status	5.2487	1.4872	0.6978			
**Perceived behavior control**	**5.6226**	**1.4009**		**0.9594**	**0.8566**	**0.9598**
It is my responsibility to buy nutritious food for my family	5.5996	1.4267	0.9014			
It is my responsibility to choose foods that make my family healthy	5.6420	1.3453	0.9402			
It is my responsibility to ensure a frequent supply of fruits to my family	5.6049	1.3976	0.9322			
I should make sure I eat healthy so that I have the energy to take care of my family	5.6437	1.4341	0.9278			
**Intention to choose health food**	**5.0780**	**1.5078**		**0.8941**	**0.7103**	**0.9052**
Suppose there are several nutritious foods to buy now; what is the possibility that you will buy more than one of them in your next market visit?	4.9400	1.5026	0.8940			
Suppose there is a variety of food to buy now, food that improves your health; what is the possibility that you will buy them in your next market visit?	4.9365	1.4716	0.9348			
Suppose there are fruits and vegetables to buy now; what is the possibility that you will buy them on your next market visit?	4.9594	1.4814	0.9008			
Suppose you have sufficient income; what is the possibility that you choose to buy nutritious food?	5.4762	1.5758	0.5974			

### 4.3 Validity and reliability of the measurement model

Appendices 2 and 3 present the measurement model test results, which were validated using the factor loadings, Cronbach's Alpha value, AVE, and CR. Values of all factor loadings were positive, and ranged from 0.5 to 0.9, which is within the accepted range of >=0.5 (51, 52), implying that the indicators strongly predict the constructs they are measuring (Table 2). It is suggested that factor loadings of indicators should be positive, and the closer the value is to 1, the stronger the estimation of the construct (71). The convergent validity of the measurement model is also met, as indicated by the values of AVE and CR, which are >0.5 and 0.6 (cut-off points), respectively (Table 2), which are accepted (48, 65). This implies that there is assurance that the constructs are well measured by their indicators. The recorded large values of factor loadings also confirm the convergent validity (52).

Furthermore, the findings show that the measurement model's discriminant validity is met because the square roots of the AVE values for each construct are greater than the correlation coefficients among the respective constructs (Table 3). This implies that there is no relationship between indicators of different constructs, and the measurement model is free from redundant indicators (49). Therefore, it is plausible to conclude that the overall measurement model proves adequate validity and reliability for estimating the structural model.

**Table 3:** Square root of the average variance extracted (AVE) and correlations of constructs.

**Constructs/AVE**	**Knowledge of healthy food**	**Attitude toward healthy eating**	**Perceived behavioral control**	**Intention to choose health food**
Knowledge of healthy food	**0.848**			
Attitude toward healthy eating	0.777	**0.8142**		
Perceived behavioral control	0.556	0.5533	**0.9255**	
Intention to choose health food	0.41	0.4333	0.446	**0.843**

The bolded values are the AVE values. Note that for each latent variable, they are higher than the correlation coefficients among the respective latent variables, indicating that discriminant validity is satisfied.

### 4.4 Influences of consumers' attitudes, perceived behavior control, and knowledge on the intention and actual consumption of healthy foods

As shown in Table 4, a significant (*p* < 0.05) positive correlation exists among all constructs of the TPB. Knowledge of healthy food exhibits a strong correlation with attitude toward healthy eating (*r* = 0.77), followed by perceived behavior control with knowledge (*r* = 0.56) and attitude (*r* = 0.55), respectively. Further, the intention to choose healthy food correlates with all other constructs, though the strength of the relationship is relatively low. Even though the correlations do not communicate any causal relationship between the constructs, they suggest the direction of the relationship, which helps to predict the potential path specification.

**Table 4:** Correlates of the latent variables.

**Constructs**	**Knowledge of healthy food**	**Attitude toward healthy eating**	**Perceived behavioral control**	**Intention to choose health food**
Knowledge of healthy food	1			
Attitude toward healthy eating	0.7771^***^	1		
Perceived behavioral control	0.5562^***^	0.5533^***^	1	
Intention to choose health food	0.4104^***^	0.4333^***^	0.4460^***^	1

^***^, ^**^, and ^*^ imply significance at *p* < 0.01, *p* < 0.05, and *p* < 0.1, respectively.

The Goodness of Fit (GoF) indices suggest that the TPB model adequately fits into the data. Due to the plethora of goodness of fit indices, Hu and Bentler (53) suggested the two-index presentation strategy of the GoF indices, which are TLI and SRMR; RMSEA and SRMR; and CFI and SRMR. With their cutoff values in parentheses, the SRMR = 0.055 (< 0.08) shows that the model was adequately specified; TLI = 0.957 (>0.90) shows that the model fitted well the data independently from the sample size; CFI = 0.964 (≥0.90) indicates the extent of variance accounted for in a covariance matrix, and the RMSEA = 0.066 (< 0.08) indicating a reasonable the model fit (48, 51, 54).

The latent variables, namely, knowledge, attitude, and perceived behavioral control, account for 93% of the variance in the consumers' intention to choose healthy foods (Table 5). Consumer attitude and perceived behavioral control serve a dual role: they directly influence the intention to choose healthy foods and mediate the effect of consumer knowledge on healthy eating. Consumer attitude (β = 0.24, *p* < 0.01) positively influences the intention to consume healthy foods. Thus, we fail to reject the hypothesis that “the consumer's attitude toward food significantly and positively predicts healthy food choice intention.” This implies that consumers consider eating healthy, especially fruits and vegetables, as good behavior, and thus, they would likely intend to perform such behavior to have good eating behavior.

**Table 5:** Influence of the constructs of the TPB on the consumers' intention and actual consumption of healthy foods.

**Causal relationship (*N* = 714)**	**Path coefficient**	**Standard error**	**[95% confidence interval]**	
Knowledge -> attitude	0.8707^***^	0.0148	0.8416	0.8997
Attitude -> perceived behavioral control	0.3723^***^	0.0912	0.1936	0.5509
Knowledge -> perceived behavioral control	0.2756^***^	0.0913	0.0966	0.4546
Attitude -> intention to consume healthy foods	0.2403^**^	0.1053	0.0339	0.4466
Perceived behavioral control -> intention to choose healthy food	0.2372^***^	0.0515	0.1362	0.3381
Knowledge -> intention to choose healthy food	0.0493	0.1037	−0.154	0.2526
Intention -> ln (fruits consumption)	0.1436^***^	0.0481	0.0494	0.2378
Intention -> ln (vegetable consumption)	0.0948^**^	0.0486	0.0005	0.1901
**Fit statistic**				
Root Mean Squared Error of Approximation (RMSEA)				0.066
Akaike's Information Criterion (AIC)				19,369.100
Bayesian Information Criterion (BIC)				19,613.896
Comparative Fit Index (CFI)				0.964
Tucker-Lewis Index (TLI)				0.957
Standardized Root Mean Squared Residual (SRMR)				0.055
R-square				0.93

^***^, ^**^, and ^*^ imply significance at *p* < 0.01, *p* < 0.05, and *p* < 0.1, respectively.

Our findings are consistent with some previous studies on consumers' food choices. A study conducted by Singh and Verma (18) in India found that a positive attitude toward organic food influences the purchase intention of organic food. This corresponds to prior research such as Nystrand and Olsen (30) on functional food consumption, Seo et al. (77) on processed foods, Miguel et al. (44) on fruit and vegetable consumption, and Donahue (55) on organic food. However, Sajjad et al. (43) found that attitude had no significant influence on fast-food consumption among college students in Pakistan. This variation in results attests to the significance of context-specific inquiry into drivers of consumer food choices.

Furthermore, the results indicate that perceived behavior control has a positive influence (β = 0.24, *p* < 0.01) on the intention to choose healthy foods; thus, the hypothesis that “perceived behavioral control significantly and positively predicts consumers' intention to choose healthy foods” is supported. This finding suggests that consumers perceive themselves as capable of consuming healthy foods and are therefore more likely to do so, indicating that they are confident that they possess the necessary resources, abilities, and skills to access healthy food. A study by Ajzen (33) found that perceived behavioral control is the strongest predictor of intentions to eat a healthy diet, and that low perceived control may reflect actual control. These findings correspond with findings of other studies that have shown a positive influence of perceived behavior control on; fast food consumption intention (56), adults' healthy eating intention (31), sustainable food consumption (32), and purchase intention of fruits and vegetables (44).

Although knowledge about healthy food was found to have no direct significant influence on healthy food choice intention, it is mediated by attitude (β = 0.87, *p* < 0.01) and perceived behavioral control (β = 0.27, *p* < 0.01). In this case, we fail to reject the hypotheses that “knowledge about healthy food significantly fosters a positive attitude toward the consumption of a healthy diet” and “knowledge about healthy food significantly influences perceived behavioral control.” This result reveals that attitude is strongly predicted by knowledge, which, according to Contento (8), can be modified through education. A study by Singh and Verma (18) found that attitude toward organic food is influenced by knowledge of organic food in India. Moreover, Contento (8) argued that knowledge is a prerequisite for healthy eating intention. This points out the importance of food and health literacy in making informed food choice decisions. For example, knowledge about the food groups that compose a healthy diet, the required share from each group in a diet, and how to meet a healthy diet with a low budget could nudge consumers toward making healthy food choices. Therefore, having the right knowledge about healthy eating can influence consumers' attitudes toward healthy eating behaviors, which can affect their intention and actual performance of these behaviors.

The results of this study further divulge that the consumer's intention to choose healthy foods was translated into actual consumption of fruits (β = 0.14, *p* < 0.001) and vegetables (β = 0.09, *p* < 0.05) thus supporting the hypothesis that “consumer's positive intention to choose healthy foods is associated with the actual consumption of healthy foods.” This implies that the consumption of fruits and vegetables in the study area significantly increased with their intention to choose healthy foods, confirming that intention predicts behavior. This result suggests that consumers perceive that they have strong control over their healthy eating behavior and can overcome barriers against performing that behavior. These findings suggest that intervening in the determinants of healthy food choice intention could increase the intentions and actual consumption of healthy foods.

Behind the consumer's attitude, knowledge, and perceived behavior control within the TPB framework, there are background factors that can influence intention, and/or actual healthy eating behavior (33). According to the findings of this study (Table 6), the consumer's income (β = 0.0552, *p* < 0.01) and gender (β = 0.5193, *p* < 0.01) significantly influence the consumers' intention to choose healthy foods. The actual consumption of fruits is significantly influenced by income (β = 0.1416, *p* < 0.01), age (β = −0.0252, *p* < 0.1), location (β = −1.0131, *p* < 0.01) and gender (β = 1.3003, *p* < 0.01), whereas the vegetable consumption is significantly influenced by age (β = 0.0249, *p* < 0.05), location (β = 0.7706, *p* < 0.01), and gender (β = −0.5638, *p* < 0.05).

**Table 6:** Influence of background factors on the intention and actual consumption of fruits and vegetables.

**Background factors**	**Intention (*****R***^**2**^ = **08.28%**, ***N*** = **328)**				**Fruits (*****R***^**2**^ = **04.44%**, ***N*** = **328)**				**Vegetables (*****R***^**2**^ = **13.33%**, ***N*** = **328)**			
	**Coefficient**	**Std. error**	**95% CI**		**Coefficient**	**Std. error**	**95% CI**		**Coefficient**	**Std. error**	**95% CI**	
Income (TZS' 100,000)	0.0552^***^	0.0203	−0.0153	0.095	0.1416^***^	0.0349	0.0728	0.2104	−0.0097	0.0290	−0.0666	0.0475
Household size	−0.0076	0.0282	−0.0631	0.0479	0.0668	0.0487	−0.0290	0.1627	−0.0188	0.0405	−0.0984	0.0608
Age	0.0125	0.0081	−0.0034	0.0284	−0.0252^*^	0.0139	−0.0527	0.0022	0.0249^**^	0.0116	0.0022	0.0478
Education	0.2909	0.1946	−0.0919	0.6737	0.5353	0.3359	−0.1256	1.1962	−0.3467	0.2789	−0.8955	0.2021
Location (Urban = 1)	0.2701	0.1955	−0.1144	0.6547	−1.0131^***^	0.3375	−1.6771	−0.3491	0.7706^***^	0.2802	0.2193	1.3219
Employment status	−0.1162	0.1871	−0.4843	0.2518	−0.2811	0.3229	−0.9166	0.3543	0.0668	0.2682	−0.4609	0.5944
Sex (Female = 1)	0.5193^***^	0.1827	0.1599	0.8787	−1.3003^***^	0.3153	−1.9207	−0.6799	−0.5638^**^	0.26184	−1.0789	−0.0486
Constant	3.7898	0.5124	2.7816	4.7979	5.5083^***^	0.0349	3.7677	7.2488	4.9499	0.7346	3.5047	6.3952

^***^, ^**^, and ^*^ imply significance at *p* < 0.01, *p* < 0.05, and *p* < 0.1, respectively.

According to the findings of this study, both the intention to choose healthy foods and the actual consumption of fruits increase with income. However, the consumption of vegetables does not change significantly with the increase in income. This suggests that higher-income consumers develop a stronger intention to choose healthy foods, which subsequently leads to greater fruit consumption. Similarly, Fernqvist et al. (10) found that income enhances economic access to healthy foods, indicating that economic empowerment can contribute to consumers' healthier food choices. Contrary to some previous studies (57, 78), the results in Table 6 indicate that female individuals show significantly higher intention to choose healthy foods; however, they consume significantly less fruits and vegetables than male individuals. This indicates that the female individuals' stated preferences and revealed preferences for healthy eating do not converge, suggesting that while they may have good intentions to eat healthily, they face challenges in putting these intentions into practice. While Ajzen (33) highlights that this intention–behavior gap can be caused by forgetting to perform a behavior or the consumer's change of mind, it is worth noting that this disparity can be attributed to gender imbalances, as in many African cultures, men often have greater economic and social privileges than women. Addressing this gender gap in healthier food consumption can play a crucial role in promoting consumers' healthier food choices.

The findings of this study further reveal that urban living is associated with a higher intention to choose healthy foods and actual consumption of vegetables, but lower fruit consumption as compared to rural living. This rural–urban disparity may be attributed to crop seasonality, as fruits are more season-sensitive than vegetables, which could lead to a more limited fruit supply in urban areas compared to rural areas during the off-season. This consumption pattern is congruent with the findings of Sandri et al. (34). Additionally, this study finds that vegetable consumption tends to increase with age, while fruit consumption decreases. The majority of studies, for example, Frehner et al. (35), have found a positive relationship between age and the consumption of healthy foods. The unexpected negative relationship between age and fruit consumption may be due to the economic accessibility of fruits, as their prices, particularly during the off-season, are often higher than those of vegetables, making them less affordable for older individuals.

## 5 Conclusion and recommendations

This study has divulged that both psychological and sociodemographic aspects of the personal food environment play a significant role in shaping consumer food choices. The consumers' knowledge about healthy eating is found to be central to the formation of a positive attitude and perceived behavior control in predicting the intention to choose healthy foods and actual consumption of fruits and vegetables. Thus, interventions designed to improve consumers' skills and knowledge about healthy eating can foster positive attitudes and perceived behavior control toward healthy eating, thereby nudging consumers to healthy lifestyles. Additionally, sociodemographic factors such as income, age, gender, and location are found to substantially impact food choices. Notably, it is important to highlight that while female individuals express a strong intention to consume healthy foods, this intention does not align with their actual consumption, as they tend to over-report their intentions but consume fewer healthy foods than their male counterparts. This discrepancy underscores the need for strategies to address the gender gap between food-related intentions and actual behaviors. Overall, health and nutrition-sensitive interventions should integrate the socio-psychological aspects of the food environment to improve consumers' perceptions and foster positive attitudes about healthy eating. Despite the valuable insights and robust validity and reliability checks that the findings of this study offer, its generalization may be subject to respondents' self-report bias and a narrow focus on the actual consumption of fruits and vegetables. Future research could validate the scale used to capture the consumer food choice behavior and explore the personal food environment influences on the intention and actual consumption of other food groups than fruits and vegetables. Furthermore, further studies should investigate other socio-psychological factors that were not captured in this study.

## Data Availability

The raw data supporting the conclusions of this article will be made available by the authors, without undue reservation.
